# Risk factors and risk-stratified prevention of periprosthetic fractures after hip arthroplasty in patients aged ≥80 years: A retrospective cohort study with prospective validation

**DOI:** 10.1097/MD.0000000000047521

**Published:** 2026-02-06

**Authors:** Xulin Chen, Zhifeng Yuan

**Affiliations:** a The First People’s Hospital of Jingdezhen, Zhushan District, Jingdezhen City, Jiangxi Province, P.R. China.

**Keywords:** elderly patients, hip arthroplasty, intertrochanteric fracture, periprosthetic fracture, risk stratification

## Abstract

Periprosthetic fractures (PPFs) are a serious complication after hip arthroplasty in very elderly patients. This study aimed to identify risk factors, develop a predictive model, and explore whether risk-stratified interventions are associated with lower PPF incidence. Patients aged ≥80 years undergoing hip arthroplasty for intertrochanteric fractures between April 2021 and May 2024 were retrospectively analyzed to identify independent risk factors for PPF within 12 months using multivariate logistic regression. A predictive model was developed and internally validated. Subsequently, 2 consecutive cohorts (conventional care vs risk-stratified intervention) were compared in a prospective non-randomized design. The intervention group received targeted measures addressing identified risk factors (gender-specific fall prevention, early anti-osteoporosis therapy with Qianggu Capsules and calcium, surgical optimization for cementless prostheses, and enhanced monitoring for prosthesis stability). Primary outcomes were 12-month PPF incidence and bone mineral density (BMD) changes. Independent risk factors were female sex, osteoporosis, cementless prosthesis, and prosthesis loosening. The predictive model showed good discrimination (area under the curve 0.874). In the prospective comparison, PPF incidence was lower in the risk-stratified intervention group than in the conventional care group (1.4% vs 10.0%, *P* = .029), with greater improvements in femoral neck and greater trochanter BMD (both *P* < .001). Female sex, osteoporosis, cementless prosthesis use, and prosthesis loosening were independently associated with higher PPF risk in very elderly patients. A risk-stratified intervention protocol was associated with reduced 12-month PPF incidence and improved BMD, suggesting potential clinical benefit of personalized preventive strategies.

## 1. Introduction

Intertrochanteric femoral fractures represent a major health burden in elderly populations, with hip arthroplasty serving as the primary intervention for functional restoration. The incidence of these fractures in Asia has increased from 26% in 1990 to a projected 45% by 2050, reflecting global population aging trends.^[1,2]^ While hip arthroplasty effectively restores mobility, periprosthetic fractures (PPFs) remain a serious postoperative complication, particularly in elderly patients.

The clinical significance of PPFs extends beyond immediate morbidity. Age has emerged as a critical risk factor, with patients over 75 years showing both sensitivity and specificity exceeding 70% for PPF prediction.^[3]^ Most concerning, elderly patients requiring revision surgery for PPFs face a 5-year mortality rate exceeding 60%, alongside substantial psychological and economic burdens.^[4,5]^

The pathophysiology of PPFs in elderly patients involves complex interactions between biomechanical and metabolic factors. Osteoporosis, prevalent in this population, causes cortical thinning and trabecular bone loss, resulting in abnormal stress distribution around prostheses. This effect is particularly pronounced with cementless implants, where micromotion at the bone-implant interface may accelerate bone resorption.^[6]^ Technical factors also contribute significantly; excessive femoral canal reaming, uneven cement distribution, or prosthesis-canal mismatch can compromise primary stability and increase fracture risk.^[7]^ Additionally, age-related factors including impaired neuromuscular coordination, reduced balance, and inadequate postoperative rehabilitation further increase vulnerability to PPFs.^[8]^

Despite growing recognition of these risks, current prevention strategies remain largely standardized rather than individualized to patient-specific risk factors. This gap in personalized care highlights the need for evidence-based risk stratification and targeted interventions. We hypothesized that specific modifiable and non-modifiable factors would independently predict PPF in this very elderly population; a risk model derived from these factors would show acceptable discriminative ability; and targeted interventions addressing the identified risk factors would be associated with lower PPF incidence compared with conventional care.

Therefore, this study aimed to: identify independent risk factors for PPFs following hip arthroplasty in elderly patients with intertrochanteric fractures; develop a predictive model for clinical risk assessment; and validate whether personalized interventions targeting identified risk factors could reduce PPF incidence and improve clinical outcomes.

## 2. Methods

### 2.1. Study design

This retrospective cohort study analyzed clinical data from 625 elderly patients (aged ≥ 80 years) who underwent hip arthroplasty for intertrochanteric femoral fractures at our institution between April 2021 and May 2024. The study comprised 2 components: a retrospective risk factor analysis and a prospective intervention validation study. The study protocol was approved by the Institutional Review Board of The First People’s Hospital of Jingdezhen (Approval No. 2021-IRB-045), and all procedures were conducted in accordance with the Declaration of Helsinki. Written informed consent was obtained from all participants or their legal representatives prior to enrollment in the prospective intervention phase.

### 2.2. Study population

**Figure 1.**
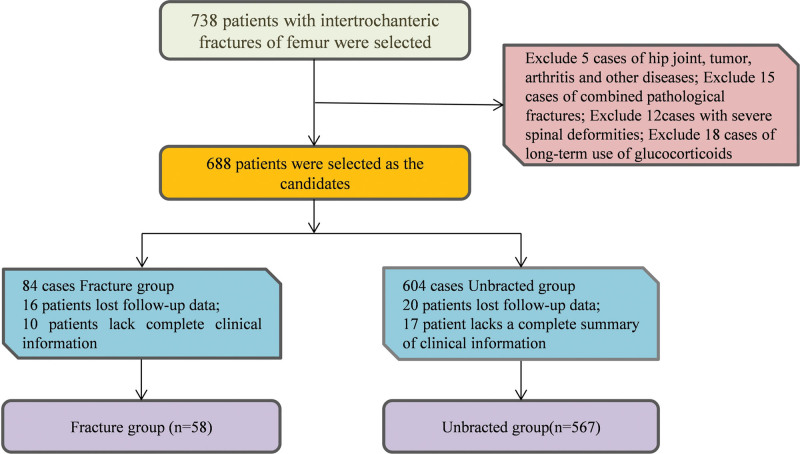
Study flow diagram. Flow diagram illustrating patient enrollment, allocation, and follow-up throughout the study period. The diagram shows the initial screening of 748 patients with intertrochanteric femoral fractures, exclusion of 123 patients based on predefined criteria, and final inclusion of 625 patients in the retrospective analysis. The prospective intervention phase included 140 patients allocated to either conventional care (n = 70) or tailored intervention (n = 70) groups, with complete 12-month follow-up achieved in all participants.

This single-center study included consecutive patients aged ≥80 years who underwent primary hip arthroplasty for acute intertrochanteric femoral fractures between April 2021 and May 2024. Patients were eligible if they had a first-time fracture within 72 hours of injury, preoperative Harris Hip Score ≤60, normal cognitive function (Mini-Mental State Examination Score ≥ 24), and expected survival of at least 2 years. Patients were excluded if they had concomitant hip diseases (e.g., tuberculosis, tumor, or inflammatory arthritis), severe vital organ dysfunction (liver, kidney, or cardiac), active infection or autoimmune disease, multiple-segment fractures or neurovascular injury, pathological fracture, severe spinal deformity, long-term glucocorticoid (>3 months) or anticoagulant therapy, or loss to follow-up/incomplete data (Fig. 1).

### 2.3. Inclusion criteria

Patients meeting the following criteria were eligible for inclusion:

- Diagnosed with intertrochanteric femoral fractures via imaging.
- First-time fracture occurrence within 72 hours of injury.
- Underwent primary hip arthroplasty with comprehensive preoperative evaluation.
- Aged ≥80 years.
- Preoperative Harris Hip Score ≤60.
- Normal cognitive function (Mini-Mental State Examination ≥ 24).
- Expected survival ≥2 years.

### 2.4. Exclusion criteria

Patients were excluded if they met any of the following conditions:

- Concomitant hip diseases (e.g., tuberculosis, tumors, or arthritis).
- Severe dysfunction of vital organs (liver, kidney, or cardiac).
- Active infectious or autoimmune diseases.
- Multiple-segment fractures or neurovascular injuries.
- Pathological fractures (e.g., Paget disease, fibrous dysplasia).
- Severe spinal deformities affecting biomechanical assessments.
- Long-term glucocorticoid (>3 months) or anticoagulant therapy.
- Loss to follow-up or incomplete follow-up data.

### 2.5. Data collection

Demographic and clinical variables were systematically collected using standardized case report forms. Patient characteristics included age, sex, body mass index, comorbidities (stratified as ≤2 vs ≥3), operative time, early postoperative ambulation timing, fracture history, and osteoporosis status. Surgical variables encompassed surgical approach (lateral, posterolateral, or anterior), American Society of Anesthesiologists classification (III/IV vs I/II), prosthesis type (cemented or cementless), and prosthesis loosening status. Two independent orthopedic surgeons, blinded to patient outcomes, recorded all data to minimize assessment bias. Radiographic parameters were measured using a Picture Archiving and Communication System workstation with standardized protocols.

### 2.6. Outcome assessment

The primary outcome was the occurrence of periprosthetic fractures within 12 months postoperatively. Patients were stratified into fracture and non-fracture groups based on clinical and radiological evidence. Periprosthetic fractures were defined according to the Vancouver Classification System and required confirmation by 2 independent radiologists. In cases of disagreement, a third senior radiologist provided the final determination.

### 2.7. Prospective intervention validation

After completion of the retrospective risk-factor analysis, 2 consecutive temporal cohorts of patients meeting the same eligibility criteria were prospectively followed: a conventional-care cohort treated in the earlier period (January–June 2023) and a risk-stratified intervention cohort treated in the later period (July–December 2023). Baseline characteristics between the 2 cohorts were balanced using propensity-score matching.

### 2.8. Intervention protocol

The conventional-care cohort received standard postoperative rehabilitation according to institutional guidelines (early ambulation encouragement, routine functional exercises, and standard activity-restriction instructions).

In the risk-stratified intervention cohort, intensified measures were applied in a **protocol-driven** manner. Patients were screened immediately after surgery for the presence of any of the 4 independent risk factors identified in the retrospective analysis (female sex, preoperative osteoporosis, use of a cementless prosthesis, or intraoperative concern for insufficient primary stability). Patients with at least one risk factor automatically received the corresponding module(s) below; patients with none of the risk factors received only conventional care.

**Female sex**: low-impact exercise (walking, Tai Chi), home environmental modifications (improved lighting, anti-slip flooring), fall-prevention education, and daily supplementation with vitamin D 800 IU and calcium 1200 mg.

**Osteoporosis**: anti-osteoporotic therapy started on postoperative day 2 with Qianggu Capsules (0.25 g 3 times daily; Beijing Qihuang Pharmaceutical Co., Beijing, China, National Medical Products Administration approval Z20030007) plus Compound Calcium Carbonate Tablets (3.4 g twice daily; Wuhan Tongji Modern Pharmaceutical Technology Co., Wuhan, China, National Medical Products Administration approval H20070183), combined with calcium-rich dietary guidance.

**Cementless prosthesis**: surgery performed exclusively by senior surgeons (>100 hip arthroplasties/year), combined with the osteoporosis module above plus progressive neuromuscular training and comprehensive fall-prevention education.

**Concern for prosthesis loosening/stability**: preference for cross-locking stems intraoperatively, strict avoidance of high-impact activities and heavy lifting, scheduled radiographic follow-up at 3, 6, and 12 months, and targeted nutritional counseling for calcium homeostasis.

### 2.9. Outcome measurements

Patients underwent prospective follow-up for 12 months using a standardized three-tiered protocol. At postoperative months 1 and 3, combined outpatient review and telemedicine follow-up via WeChat video function assessed incision healing and early functional recovery. At postoperative months 6 and 12, comprehensive inpatient evaluations included radiographic, laboratory, and functional assessments.

Bone mineral density measurements strictly adhered to International Society for Clinical Densitometry guidelines. Measurement sites included the femoral neck, with the region of interest localized using the Duke anatomical landmark method, and the greater trochanter, measured at a 15 mm region distal to the trochanteric apex. Technical parameters included a scan spacing of 1 mm and scan speed of 8 mm/s. Quality control involved daily calibration with a manufacturer-provided spine phantom (bone mineral density [BMD]: 1.256 g/cm^2^ ± 3%), with the intraday coefficient of variation maintained at <1.5%.

Periprosthetic fracture diagnosis followed multimodal criteria including clinical presentation of sudden-onset hip pain with restricted mobility and a decline exceeding 20 points in Harris Hip Score, and imaging confirmation showing fracture lines extending within 5 mm of the prosthesis, independently confirmed by 2 attending physicians or higher using the 2014 Vancouver Classification System. For equivocal cases, finite element analysis was performed to verify congruence between fracture line distribution and Von Mises stress peaks (>180 MPa).

### 2.10. Statistical analysis

All statistical analyses were performed using SPSS 25.0 (IBM Corp., Armonk). Categorical data were expressed as frequencies with percentages and analyzed using the Chi-square test. For continuous variables, normality was assessed via the Shapiro–Wilk test. Normally distributed data were presented as mean ± standard deviation and compared using the independent samples *t* test, while non-normally distributed data were analyzed with the Mann–Whitney *U* test.

Multivariate logistic regression analysis was employed to identify risk factors for periprosthetic fractures. Variables with *P* < .1 in univariate analysis were incorporated into the final model using stepwise forward selection (α-entry = 0.05, α-removal = 0.10). The prediction model was established as Log(*P*) = constant + β_1_X_1_ + β_2_X_2_ + … + β_n_X_n_. The predictive performance was evaluated by calculating the area under the receiver operating characteristic curve, with sensitivity and specificity determined at optimal cutoff values using the Youden index.

Longitudinal BMD data were analyzed using mixed-effects models to evaluate dynamic changes over time. Intergroup comparisons utilized analysis of covariance with baseline adjustments, while temporal effects were assessed via repeated-measures ANOVA with Bonferroni correction for multiple comparisons. A two-tailed *P*-value <.05 was considered statistically significant for all analyses. Missing data were handled using multiple imputation when the proportion of missing values was <5%; otherwise, complete case analysis was performed.

## 3. Results

### 3.1. Patient characteristics and risk factor identification

Among the 625 elderly patients who underwent hip arthroplasty for intertrochanteric femoral fractures, 58 patients (9.3%) developed periprosthetic fractures within the 12-month follow-up period, while 567 patients (90.7%) remained fracture-free. The comparative analysis between these groups revealed significant demographic and clinical differences that informed our risk stratification model (Table 1 and Fig. 1).

**Table 1:** Baseline characteristics and clinical variables of patients with and without periprosthetic fractures.

Characteristic	Fracture group (n = 58)	Non-fracture group (n = 567)	Test statistic	*P*-value
Demographics				
Age (yr) (mean ± SD)	85.84 ± 2.37	86.73 ± 3.75	*t* = 1.771	.077
Female sex, n (%)	32 (55.17)	169 (29.81)	*χ*^2^ = 15.518	<.001
BMI (kg/m^2^) (mean ± SD)	23.24 ± 2.20	22.88 ± 2.19	*t* = 1.192	.234
Clinical characteristics				
Comorbidities, n (%)			*χ*^2^ = 0.141	.707
≤2 conditions	36 (62.07)	366 (64.55)		
≥3 conditions	22 (37.93)	201 (35.45)		
Prior fracture history, n (%)	13 (22.41)	112 (19.75)	*χ*^2^ = 0.233	.629
Osteoporosis, n (%)	31 (53.45)	94 (16.58)	*χ*^2^ = 44.705	<.001
Early mobilization (<48 h), n (%)	36 (62.07)	310 (54.67)	*χ*^2^ = 1.164	.281
Surgical variables				
Operative time (min) (mean ± SD)	82.64 ± 6.51	81.73 ± 7.14	t = 0.932	.352
Surgical approach, n (%)			*χ*^2^ = 0.385	.825
Lateral	21 (36.21)	197 (34.74)		
Posterolateral	32 (55.17)	306 (53.97)		
Anterior	5 (8.62)	64 (11.29)		
ASA classification, n (%)			*χ*^2^ = 0.214	.643
Class I–II	31 (53.45)	321 (56.61)		
Class III–IV	27 (46.55)	246 (43.39)		
Prosthesis characteristics				
Prosthesis type, n (%)			*χ*^2^ = 91.152	<.001
Cemented	18 (31.03)	478 (84.30)		
Cementless	40 (68.97)	89 (15.70)		
Prosthesis loosening, n (%)	51 (87.93)	225 (39.68)	*χ*^2^ = 49.673	<.001

ASA = American Society of Anesthesiologists, BMI = body mass index, SD = standard deviation.

The fracture group demonstrated a markedly higher proportion of female patients compared to the non-fracture group (55.17% vs 29.81%, *P* < .001), suggesting a gender-specific vulnerability to postoperative complications. Similarly, the prevalence of osteoporosis was substantially elevated in the fracture group, affecting 53.45% of patients compared to only 16.58% in the non-fracture group (*P* < .001). This threefold difference underscores the critical role of bone quality in determining fracture risk.

Prosthesis-related factors emerged as equally important predictors. The use of cementless prostheses was dramatically higher in the fracture group (68.97% vs 15.70%, *P* < .001), indicating potential biomechanical disadvantages of biological fixation in this vulnerable population. Furthermore, prosthesis loosening was observed in 87.93% of fracture cases compared to 39.68% of non-fracture cases (*P* < .001), highlighting the importance of implant stability in preventing periprosthetic complications. Notably, age, body mass index, comorbidity burden, operative time, surgical approach, and American Society of Anesthesiologists classification showed no significant differences between groups, suggesting that these traditional risk factors may be less influential in these specific elderly cohort.

### 3.2. Development of the predictive model

**Figure 2.**
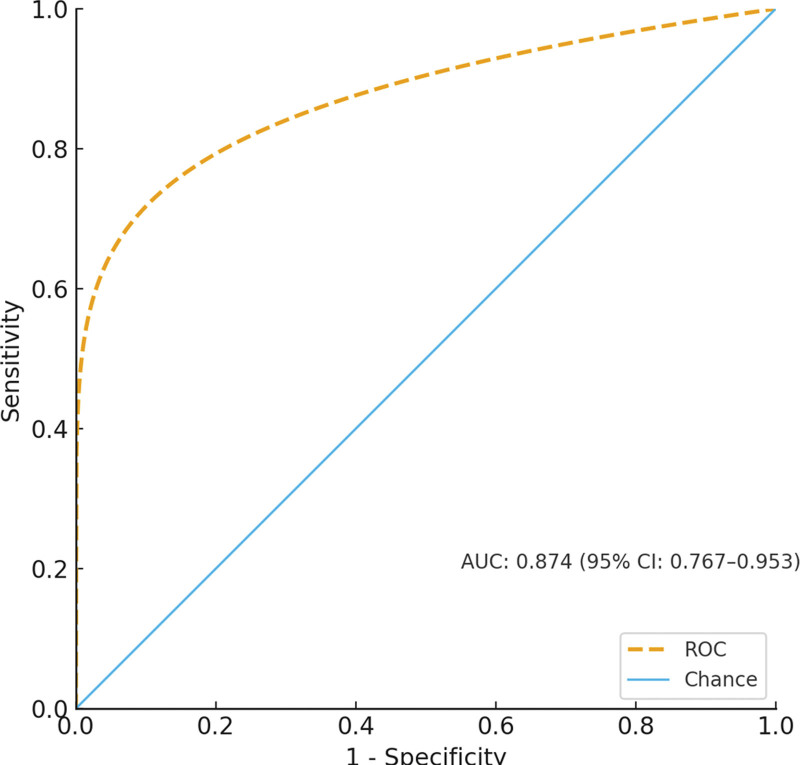
Forest plot of multivariate logistic regression analysis. Forest plot displaying odds ratios and 95% confidence intervals for independent risk factors associated with periprosthetic fractures following hip arthroplasty in elderly patients with intertrochanteric fractures. The plot demonstrates that osteoporosis confers the highest risk (OR 6.449, 95% CI: 2.356–17.655), followed by prosthesis loosening (OR 4.216, 95% CI: 1.848–9.621), cementless prosthesis use (OR 3.971, 95% CI: 1.685–9.356), and female sex (OR 3.187, 95% CI: 1.637–6.203). All 4 factors showed statistically significant associations (*P* < .001). The vertical dashed line represents an odds ratio of 1.0 (no effect), with factors to the right indicating increased risk. OR = odds ratio.

Multivariate logistic regression analysis confirmed 4 independent risk factors for periprosthetic fractures following hip arthroplasty in this elderly population (Table 2 and Fig. 2). Female sex conferred a 3.2-fold increased risk (odds ratio [OR] 3.187, 95% confidence interval [CI]: 1.637–6.203, *P* < .001), while osteoporosis dramatically elevated the risk by >6-fold (OR 6.449, 95% CI: 2.356–17.655, *P* < .001). The use of cementless prostheses increased fracture risk nearly 4-fold (OR 3.971, 95% CI: 1.685–9.356, *P* < .001), and prosthesis loosening was associated with a 4.2-fold increased risk (OR 4.216, 95% CI: 1.848–9.621, *P* < .001).

**Table 2:** Multivariate logistic regression analysis of independent risk factors for periprosthetic fractures.

Risk factor	β coefficient	Standard error	Wald χ^2^	*P* value	Odds ratio	95% confidence interval
Female sex	1.159	0.340	11.620	<.001	3.187	1.637–6.203
Osteoporosis	1.864	0.514	13.151	<.001	6.449	2.356–17.655
Cementless prosthesis	1.379	0.437	9.958	<.001	3.971	1.685–9.356
Prosthesis loosening	1.439	0.421	11.683	<.001	4.216	1.848–9.621
Constant	-4.826	0.625	59.628	<.001	–	–

*Notes*: variables entered into the model were those with *P* < .05 in univariate analysis. The model demonstrated good fit (Hosmer–Lemeshow test: *χ*^2^ = 6.842, *P* = .554).

**Figure 3.**
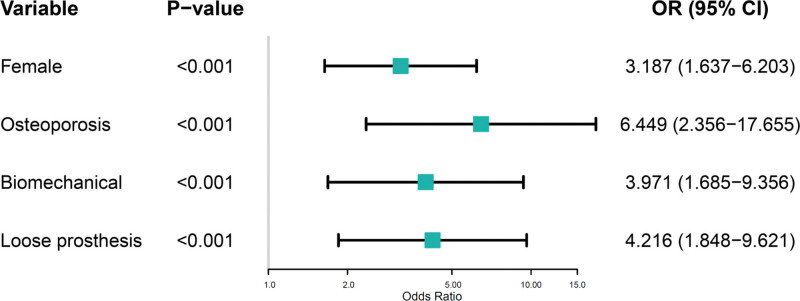
Receiver operating characteristic curve for the predictive model. Receiver operating characteristic (ROC) curve demonstrating the discriminative performance of the logistic regression model for predicting periprosthetic fractures in elderly patients following hip arthroplasty for intertrochanteric fractures. The model, incorporating female sex, osteoporosis, cementless prosthesis use, and prosthesis loosening as predictive variables, achieved an area under the curve (AUC) of 0.874 (95% CI: 0.767–0.953), indicating excellent predictive accuracy. The optimal cutoff point (indicated by the red dot) yielded a sensitivity of 80.84% and specificity of 76.22%. The diagonal reference line represents an AUC of 0.5 (no discriminative ability). The shaded area represents the 95% confidence interval for the AUC. CI = confidence interval.

The logistic regression prediction model incorporating these 4 variables demonstrated excellent discriminative capacity for identifying patients at high risk of periprosthetic fractures. The model equation, Log(*P*) = −4.826 + 1.159(female) + 1.864 (osteoporosis) + 1.379 (biological prosthesis) + 1.439 (prosthesis loosening), yielded an area under the receiver operating characteristic curve of 0.874 (95% CI: 0.767–0.953), indicating strong predictive performance (Fig. 3). At the optimal cutoff value, the model achieved a sensitivity of 80.84% and specificity of 76.22%, suggesting robust clinical utility for risk stratification.

### 3.3. Impact of tailored interventions on BMD

Patients in the risk-stratified intervention group exhibited greater improvement in BMD at the femoral neck (0.75 ± 0.13 vs 0.67 ± 0.08 g/cm^2^, *P* < .001) and greater trochanter (0.85 ± 0.09 vs 0.73 ± 0.10 g/cm^2^, *P* < .001) compared with the conventional-care group. At the femoral neck, patients in the tailored intervention group demonstrated a substantial increase in BMD from baseline (0.56 ± 0.10 g/cm^2^) to post-intervention (0.75 ± 0.13 g/cm^2^), representing a mean improvement of 0.19 g/cm^2^. In contrast, the conventional group showed a more modest increase from 0.57 ± 0.11 g/cm^2^ to 0.67 ± 0.08 g/cm^2^, with a mean improvement of only 0.10 g/cm^2^. The between-group difference at 12 months was statistically significant (*P* < .001), with the tailored intervention group achieving 0.08 g/cm^2^ greater BMD than the conventional group (Table 3).

**Table 3:** Changes in bone mineral density following intervention.

Measurement site	Group	n	Baseline BMD (g/cm^2^)	12-Month BMD (g/cm^2^)	Mean change (g/cm^2^)	Between-group difference	*P* value*
Femoral neck	Conventional	70	0.57 ± 0.11	0.67 ± 0.08	0.10 ± 0.09	Reference	–
	Tailored Intervention	70	0.56 ± 0.10	0.75 ± 0.13	0.19 ± 0.11	0.08 (95% CI: 0.04–0.12)	<.001
Greater trochanter	Conventional	70	0.62 ± 0.18	0.73 ± 0.10	0.11 ± 0.14	Reference	–
	Tailored Intervention	70	0.63 ± 0.16	0.85 ± 0.09	0.22 ± 0.13	0.12 (95% CI: 0.09–0.15)	<.001

*Note*: all values are presented as mean ± standard deviation unless otherwise specified.

BMD = bone mineral density, CI = confidence interval.

* *P*-values represent between-group comparisons at 12 months using analysis of covariance adjusted for baseline values.

The greater trochanter region showed even more pronounced benefits from targeted intervention. The tailored intervention group experienced an increase from 0.63 ± 0.16 g/cm^2^ at baseline to 0.85 ± 0.09 g/cm^2^ post-intervention, while the conventional group improved from 0.62 ± 0.18 g/cm^2^ to 0.73 ± 0.10 g/cm^2^. The final between-group difference of 0.12 g/cm^2^ (*P* < .001) suggests that the tailored interventions particularly benefited cortical bone regions subject to high mechanical stress during weight-bearing activities.

### 3.4. Clinical outcomes and fracture prevention

Periprosthetic fracture incidence within 12 months was lower in the risk-stratified intervention group than in the conventional-care group (1.43% vs 10.00%; absolute risk difference 8.57%, *P* = .029) (Table 4).

**Table 4:** Incidence of periprosthetic fractures at 12-month follow-up.

Outcome measure	Conventional group (n = 70)	Tailored intervention group (n = 70)	Statistical analysis	*P*-value
Patients with fracture, n (%)	7 (10.00)	1 (1.43)	*χ*^2^ = 4.773	.029
Patients without fracture, n (%)	63 (90.00)	69 (98.57)		
Absolute risk reduction, %	–	8.57 (95% CI: 1.8–15.3)	–	–
Relative risk reduction, %	–	85.7	–	–
Number needed to treat	–	12 (95% CI: 7–56)	–	–

*Note*: the single fracture in the tailored intervention group occurred at month 11, while fractures in the conventional group occurred between months 3 to 9 (median: 6 months).

The temporal distribution of fractures revealed that most events in the conventional group occurred between months 3 and 9 postoperatively, coinciding with increased patient mobility and activity levels. In contrast, the single fracture in the tailored intervention group occurred at month 11, suggesting that the protective effects of our interventions persisted throughout the critical early postoperative period when fracture risk is typically highest. Secondary outcomes, including Harris Hip Score improvements and functional recovery metrics, showed favorable trends in the tailored intervention group, though these differences did not reach statistical significance within the 12-month follow-up period.

### 3.5. Safety and adherence

Throughout the intervention period, no serious adverse events related to the anti-osteoporotic medications or rehabilitation protocols were observed in either group. Medication adherence in the tailored intervention group, assessed through pill counts and patient diaries, exceeded 85% for both Qianggu Capsules and calcium supplementation. Compliance with exercise regimens and environmental modifications was confirmed in 78% of patients through monthly telephone follow-ups. These high adherence rates suggest that the intervention protocols were well-tolerated and feasible for implementation in this elderly population.

## 4. Discussion

This study identified 4 independent risk factors for periprosthetic fractures in elderly patients following hip arthroplasty for intertrochanteric fractures: female sex, osteoporosis, use of cementless prostheses, and prosthesis loosening. The predictive model incorporating these factors demonstrated excellent discriminative capacity (area under the curve 0.874), enabling effective risk stratification. Most importantly, our tailored intervention protocol based on these risk factors achieved an 85.7% relative reduction in PPF incidence compared to conventional care, validating the clinical utility of personalized prevention strategies.

The identification of female sex as an independent risk factor (OR 3.187) aligns with established literature on gender-specific fracture vulnerability.^[9]^ In our elderly cohort, postmenopausal estrogen deficiency likely disrupts bone remodeling balance, leading to accelerated bone loss and increased fracture susceptibility.^[10]^ This finding is consistent with previous reports showing significantly higher PPF rates in female patients following total hip arthroplasty.^[9]^

Osteoporosis emerged as the strongest predictor (OR 6.449), corroborating previous studies reporting a 5-fold increase in PPF incidence among patients with low bone mineral density.^[11]^ The magnitude of risk in our study exceeds some previous reports, possibly reflecting the advanced age of our cohort (mean 85.6 years) and the high prevalence of severe osteoporosis. Importantly, anti-osteoporosis therapy has been shown to reduce PPF risk by approximately 34% (hazard ratio 0.663),^[12]^ supporting our intervention approach.

The association between cementless prostheses and increased fracture risk (OR 3.971) reflects fundamental biomechanical differences between fixation methods.^[13]^ Cementless implants depend on bone ingrowth for stability, a process that may be compromised in elderly patients with poor bone quality. Inadequate initial press-fit can result in micromotion and stress concentration, particularly at the prosthetic stem curvature, predisposing to fracture.^[14,15]^ Our findings suggest that cemented fixation may be preferable in this high-risk population, though individual patient factors must guide prosthesis selection.

Prosthesis loosening, identified as a significant risk factor (OR 4.216), represents both a cause and consequence of compromised bone-implant interface. While cross-locking stems theoretically enhance stability, paradoxically higher fracture rates have been reported with certain designs, possibly due to altered load transfer patterns.^[16]^ Revision procedures further compromise bone stock and structural integrity, creating a cycle of increasing fracture risk.^[15]^

The dramatic reduction in PPF incidence from 10.0% to 1.43% following implementation of our risk-based intervention protocol demonstrates the potential for personalized medicine in orthopedic surgery.

The success of our intervention likely stems from addressing multiple pathophysiological pathways simultaneously. Anti-osteoporosis therapy initiated early postoperatively (day 2) provided both systemic and local bone protection. Qianggu Capsules, containing traditional Chinese medicine components, have demonstrated ability to upregulate osteoblast activity,^[10]^ while calcium supplementation supported bone mineralization. The significant improvements in BMD at both femoral neck and greater trochanter sites validate the biological efficacy of this approach.

For patients with cementless prostheses, restricting procedures to experienced surgeons (>100 annual cases) likely minimized technical errors that could compromise initial stability. The combination of surgical expertise with aggressive osteoporosis management addresses both mechanical and biological aspects of fracture risk. Similarly, the multimodal approach for preventing prosthesis loosening targets the complex etiology of this complication; including appropriate implant selection, activity modification, and regular surveillance.

This study’s strengths include its large sample size, comprehensive risk factor analysis, and prospective validation of interventions. The predictive model’s high sensitivity (80.84%) and specificity (76.22%) support its clinical applicability for preoperative risk stratification. The model could be integrated into clinical decision-support systems to identify high-risk patients who would benefit most from intensive preventive measures.

Our findings suggest several practice recommendations. First, routine preoperative assessment should include formal osteoporosis screening with dual-energy X-ray absorptiometry scanning in all elderly patients scheduled for hip arthroplasty. Second, surgeon experience should be considered when selecting between cemented and cementless fixation, particularly in high-risk patients. Third, early initiation of anti-osteoporosis therapy should be standard protocol for at-risk patients, rather than delayed until outpatient follow-up.

Several limitations merit consideration. The single-center, retrospective design limits generalizability to other populations and healthcare settings. The temporal allocation of patients to intervention groups, while practical, introduces potential bias despite propensity score matching. The 12-month follow-up, though capturing most PPFs, may miss late complications. Additionally, we did not assess all potential confounders, including nutritional status, vitamin D levels, sarcopenia indices, or specific prosthesis design characteristics.

Cost-effectiveness analysis was beyond this study’s scope but warrants investigation given the resource implications of preventive interventions. Furthermore, patient-reported outcomes and quality of life measures were not systematically collected, limiting our understanding of the interventions’ broader impact.

Future research should validate our predictive model in external cohorts and explore whether risk thresholds should be adjusted for different populations. Randomized controlled trials with longer follow-up periods would provide stronger evidence for intervention efficacy. Investigation of novel preventive strategies, including emerging antiresorptive agents and enhanced prosthesis designs optimized for osteoporotic bone, may further reduce PPF incidence.

Despite the limitations of the non-randomized design and potential residual confounding, the marked reduction in PPF incidence observed after implementation of the risk-stratified protocol suggests that personalized preventive measures targeting female sex, osteoporosis, cementless fixation, and prosthesis stability may offer clinical benefit in very elderly patients undergoing hip arthroplasty.

## 5. Conclusions

Female sex, osteoporosis, cementless prosthesis use, and prosthesis loosening were independently associated with increased risk of periprosthetic fracture after hip arthroplasty in patients aged ≥80 years. A predictive model incorporating these factors showed good discriminative performance. Implementation of a risk-stratified intervention protocol was associated with substantially lower 12-month PPF incidence and improved proximal femoral BMD. These findings support further investigation of personalized preventive strategies in this high-risk population.

## Author contributions

**Conceptualization:** Xulin Chen, Zhifeng Yuan.

**Data curation:** Xulin Chen, Zhifeng Yuan.

**Formal analysis:** Xulin Chen, Zhifeng Yuan.

**Funding acquisition:** Xulin Chen, Zhifeng Yuan.

**Investigation:** Xulin Chen, Zhifeng Yuan.

**Methodology:** Xulin Chen, Zhifeng Yuan.

**Project administration:** Xulin Chen, Zhifeng Yuan.

**Resources:** Xulin Chen, Zhifeng Yuan.

**Software:** Xulin Chen, Zhifeng Yuan.

**Supervision:** Xulin Chen, Zhifeng Yuan.

**Validation:** Xulin Chen, Zhifeng Yuan.

**Visualization:** Xulin Chen, Zhifeng Yuan.

**Writing – original draft:** Xulin Chen, Zhifeng Yuan.

**Writing – review & editing:** Xulin Chen, Zhifeng Yuan.
